# Geographic Disparities in Re-triage Destinations Among Seriously Injured Californians

**DOI:** 10.1097/AS9.0000000000000270

**Published:** 2023-03-17

**Authors:** Al’ona Furmanchuk, Kelsey James Rydland, Renee Y. Hsia, Robert Mackersie, Meilynn Shi, Mark William Hauser, Abel Kho, Karl Y. Bilimoria, Anne M. Stey

**Affiliations:** From the * Division of General Internal Medicine and Geriatrics, Northwestern University Feinberg School of Medicine, Chicago, IL; † Center for Health Information Partnerships (CHiP), Institute for Public Health and Medicine, Northwestern University Feinberg School of Medicine, Chicago, IL; ‡ Northwestern University Libraries, Research and Data Services, Evanston, IL; § Department of Emergency Medicine, University of California San Francisco, San Francisco, CA; ∥ Philip R. Lee Institute for Health Policy Studies, University of California San Francisco, San Francisco, CA; ¶ Department of Surgery, University of California San Francisco, San Francisco, CA; # Department of Anthropology, Northwestern University, Evanston, IL; ** Department of Surgery, Northwestern University Feinberg School of Medicine, Chicago, IL

**Keywords:** geospatial mapping, network analysis, optimality, re-triage, serious injury, trauma

## Abstract

**Objective:**

The objective of this study is to quantify geographic disparities in suboptimal re-triage of seriously injured patients in California.

**Summary of background data:**

Re-triage is the emergent transfer of seriously injured patients from the emergency departments of nontrauma and low-level trauma centers to, ideally, high-level trauma centers. Some patients are re-triaged to a second nontrauma or low-level trauma center (suboptimal) instead of a high-level trauma center (optimal).

**Methods:**

This was a retrospective observational cohort study of seriously injured patients, defined by an Injury Severity Score >15, re-triaged in California (2009–2018). Re-triages within 1 day of presentation to the sending center were considered. The suboptimal re-triage rate was quantified at the state, regional trauma coordinating committees (RTCC), local emergency medical service agencies, and sending center level. A generalized linear mixed-effects regression quantified the association of suboptimality with the RTCC of the sending center. Geospatial analyses demonstrated geographic variations in suboptimal re-triage rates and calculated alternative re-triage destinations.

**Results:**

There were 8,882 re-triages of seriously injured patients and 2,680 (30.2%) were suboptimal. Suboptimally re-triaged patients had 1.5 higher odds of transfer to a third short-term acute care hospital and 1.25 increased odds of re-admission within 60 days from discharge. The suboptimal re-triage rates increased from 29.3% in 2009 to 38.6% in 2018. However, 56.0% of nontrauma and low-level trauma centers had at least one suboptimal re-triage. The Southwest RTCC accounted for the largest proportion (39.8 %) of all suboptimal re-triages in California.

**Conclusion:**

High population density geographic areas experienced higher suboptimal re-triage rates.

## INTRODUCTION

Trauma systems have decreased injury-associated mortality rates since their development in the 1970s.^1,2^ Trauma systems coordinate care at each step, from prehospital field triage by emergency medical services (EMS) to definitive care provided by specialized teams at trauma centers. The Centers for Disease Control and Prevention field triage guidelines were developed to promote efficient EMS triage of seriously injured patients from the field directly to high-level trauma centers.^3^ Yet 17%–34% of seriously injured patients are still under-triaged from the field to nontrauma or low-level trauma centers.^4,5^ Seriously injured patients who are under-triaged have a 30% higher likelihood of mortality during the 48 hours after injury.^6^ Re-triage is the emergent transfer of under-triaged, seriously injured patients from an emergency department (ED) of a nontrauma or low-level trauma center to a high-level trauma center. Studies have shown those seriously injured patients re-triaged within 2 hours have equivalent mortality to those who are field triaged directly to a high-level trauma center.^7,8^

However, in practice, re-triage occurs too slowly with a median time of 4 hours.^9^ Re-triage time is affected by the presence of a well-defined re-triage process where sending centers recognize an injured patient’s condition, adhere to geographic-specific re-triage guidelines if they exist, as well as sufficient coordination of resources between sending and receiving centers to complete transport. State-wide trauma system coordination in California is highly decentralized with 33 local EMS agencies (LEMSAs) administering services across 58 counties, with limited oversight by the California Emergency Medical Services Authority. Some LEMSAs have clear re-triage guidelines and strong coordination among hospitals, whereas others may not. We anticipated that de-centralized coordination could lead to geographic variation in suboptimal re-triage, where patients at nontrauma or low-level trauma centers may be sent to a second nontrauma or low-level trauma center rather than to a high-level trauma center. Yet, little is known about geographic variation and disparities in suboptimal re-triage in California.

The main objective of this analysis was to understand geographic disparities in the sub-optimal re-triage of seriously injured patients (Injury Severity Score^10^ [RISS] >15) over the past decade in California. The first aim was to quantify suboptimal re-triage at the level of the state, regional trauma coordinating committees (RTCC), LEMSA, and sending center. The second aim was to compare hospital course between suboptimal and optimally re-triaged patients. The third aim was to determine if there was significant variation in suboptimal re-triage rates at the RTCC level while adjusting for patient characteristics, sending center, year, and LEMSA. The fourth aim was to determine if a more optimal alternative re-triage destination could be identified. The *a priori* hypothesis was that suboptimal re-triage would not be uniformly geographically distributed across California and could be optimized using network visualization and optimization estimations.

## METHODS

This was a retrospective observational cohort study of seriously injured adults in California who were re-triaged or emergently transferred from an ED at a nontrauma or level III/IV trauma center to a second receiving center within 1 day, as captured in linked ED and inpatient records from 2009 to 2018. We defined suboptimal re-triage as re-triage of a seriously injured patient from an ED at a nontrauma or level III/IV trauma center to a second nontrauma or level III/IV trauma center. Suboptimal re-triage prevalence was quantified at the state, RTCC, LEMSA, and sending center levels. This study was approved by the Institutional Review Board of Northwestern University (STU00211123).

### Data Source and Study Sample

We used nonpublic data from the California Department of Healthcare Access and Information (HCAI) ED and inpatient discharge datasets from 2009 to 2018. The nonpublic HCAI datasets were administrative data submitted by all hospitals for every ED and inpatient hospital encounter in the state of California annually. These data have a unique patient identifier, the record linkage number (RLN), which allows linkage across hospital encounters. Additionally, the HCAI maintains publicly accessible, comprehensive, updated hospital characteristics data, such as trauma designation and location. The California Annual Hospital Utilization Reports^11^ were merged into the nonpublic HCAI discharge data.

#### Encounter Linkage

The number of records available from the ED and inpatient files does not always represent the number of unique encounters. The number of duplicate records varied by year. All duplicate record entries were cleaned before linkage was performed. The RLN, encounter date in the sending ED, encounter date in the receiving center, and discharge disposition from the sending ED were linked to create the encounter pairs.

The annual trauma center-level designation was obtained from publicly available California Annual Hospital Utilization Reports. Seriously injured patient encounters that initially presented to the ED of a nontrauma or level III/IV trauma center and were transferred to another short-term acute care hospital within 1 day were labeled as re-triaged. These data lacked time stamps, yielding poor identification of re-triages that occur around midnight. The 1-day difference between sending discharge and receiving admission date was selected to include re-triages that started as a late-night presentation to the sending ED and ended up as early morning admissions to a receiving center.

#### Inclusion Criteria

Adult patients aged 18–89 years were included. Age range selection was defined by health privacy laws to exclude rare extreme outliers (90 and older) that may be identifiable.^12,13^ Only encounters with an injury diagnosis defined by the International Classification of Disease (ICD) codes (ICD-9: 800-904.9, 910-939.0, and 950-959.9, ICD-10: S00-T19, T33-T34, T79) presenting to nontrauma or level III/IV trauma centers between January 1, 2009, and December 31, 2018, were included. A RISS was derived from encounter ICD codes using a validated Programs for Injury Categorization (ICDPIC)-R program.^10^ The program used the R alternative to the STATA version 14.2 of ICDPIC to produce RISS. Only encounters with major injuries, as defined by a RISS >15, were included per recommendations^14^ because these patients are most likely to derive mortality benefits from re-triage.^15^ Encounters with burn injuries, as defined by the ICD codes, were not considered. Only patients initially admitted to non-trauma or level III/IV trauma centers, labeled as ‘short-term general acute care hospitals’, were included.

#### Exclusion Criteria

Interfacility transfer is the nonemergent transfer between EDs or inpatient units to a second specialized center. Interfacility transfers were identified as such when a transfer occurred more than 1 day after the initial ED presentation at the nontrauma or low-level trauma center. Interfacility transfers were excluded from the analysis. All encounters at hospitals other than short-term general acute-care hospitals, such as rehabilitation hospitals, psychiatric hospitals, and long-term care facilities, were excluded. All elective admissions were excluded. All patients who were field triaged directly to level I or II trauma centers were excluded. All patients initially taken to nontrauma or level III/IV trauma centers but discharged home, admitted, or transferred to hospitals other than short-term general acute care hospitals, such as rehabilitation hospitals, psychiatric hospitals, and long-term care facilities were excluded. ED records with discharge dispositions ‘expired’, ‘left against medical advice or discontinued care’, ‘discharged/transferred to an inpatient rehabilitation facility including rehabilitation distinct part unit of a hospital with a planned acute care hospital inpatient readmission’ were excluded. The complete data-management protocol is presented in Supplemental Table 1, http://links.lww.com/AOSO/A221.

### Variables

#### Primary Exposure

Re-triage guidelines have been drafted and implemented variably by LEMSAs across the state.^16^ LEMSAs were organized into unofficial voluntary committees, known as the RTCC. RTCC does not have the ability or authority to implement guidelines or policies. However, the California population was more equally distributed across the RTCCs compared to the LEMSAs. Additionally, RTCC was a much larger unit of analysis in which more precise estimates less likely to be biased by low counts could be calculated. Finally, the HCAI data-use agreement prevented the reporting of low counts and rare events, which could be identifiable. Therefore, RTCC was the primary exposure variable selected to capture the geographic variation in the sub-optimal re-triage rate.

#### Primary Outcome

Sub-optimal re-triage was defined as the re-triage of seriously injured patients from a nontrauma or level III/IV trauma center to a second nontrauma center or level III/IV within 1 day. Re-triages from nontrauma or level III/IV centers to any level I/II trauma center within 1 day were all labeled as optimal re-triages regardless of the distance between the sending and receiving centers. It was not possible to calculate the exact re-triage times because of the lack of sending center discharge times, receiving center admission times, and inter-hospital transportation types.

#### Co-variates of Interest

Demographic data (age, sex, race/ethnicity, and insurance status) were included as covariates because of the known disparities in field and re-triage among elderly, female, and minority patients.^17^ Age was categorized with 18–24 years as reference, and then by 10-year intervals until 89 as follows; 25–34, 35–44, 45–54, 55–64, 65–74, 75–84, and 85–89 years. Sex was categorized as male as reference, and female. Race was categorized as White, as reference, African American, Asian, Other, and Unknown. Ethnicity was categorized as Hispanic or non-Hispanic, with the latter as reference. Insurance was categorized into self-pay (as reference), Medicaid (Medi-Cal), Medicare (Health Maintenance Organization, Medicare Risk, Medicare Part A, Medicare Part B), Private (e.g., Commercial Insurance Company, Exclusive Provider Organization, Blue Cross/Blue Shield), Unspecified managed care (e.g., Health Maintenance Organization, Preferred Provider Organization, and Point of Service), and other. When a managed care category was reported, it was mapped to private insurance. However, hospitals may inadvertently include patients covered by a Medicare-managed care program administered by a private insurance company under a managed care unspecified category.^18,19^ We kept the managed care unspecified category as defined in the HCAI to avoid ambiguity rather than subsuming under Medicare, Medicaid, or private for that reason. RISS was calculated, as described above, and was categorized as RISS <25 as reference, and RISS >25. Injury mechanisms were grouped into “all transport”, as reference, “fall”, “struck by or against”, “other”, and “unspecified”. The year of re-triages, the LEMSA, and the sending hospital ID were included as covariates.

Variables that captured the hospital course at the receiving hospital center were calculated. The rate of diagnostic, minor, and major therapeutic surgery as captured by ICD procedure codes at the receiving hospital using procedure classes refind for ICD-10-PCS,^20^ and the clinical classifications software for ICD-10-PCS.^21^ The length of stay at the receiving hospital center was calculated for each re-triage as the difference between the discharge and admission date. The discharge disposition at the receiving hospital center was collapsed into 4 categories (see Supplemental Table 5, http://links.lww.com/AOSO/A221) home, died, short-term acute care hospital, and post-acute care. Finally, RLN was used to identify the occurrence of readmission of re-triaged seriously injured patients who survived to discharge at 10, 30 and 60 days.

### Statistical Analysis

Continuous data were reported as means and standard deviations. Categorical data were reported as re-triage counts and percentages. The count and rate of sub-optimal re-triage were calculated for the entire state of California. The proportion of state-wide sub-optimal re-triage was calculated for each RTCC, LEMSA, and sending center. The rate of sub-optimal re-triage was calculated for each RTCC, LEMSA, and sending center.

Patient characteristics were compared between suboptimal and optimal re-triages using a *χ*^2^ test. Hospital course including diagnostic, minor and major surgery rates, length of stay discharge dispositions, and re-admission rates were compared between suboptimal and optimal re-triages using the *χ*^2^ test. A generalized linear mixed-effects (GLMM) regression modeling the probability of suboptimal re-triage was used to quantify the association with RTCC of the sending center while controlling for a priori determined fixed-effect predictor variables including age, sex, race, ethnicity, insurance status, RISS, injury mechanism, and random effects to control for clustering by sending center and year. All data management and analyses were performed using R version 4.1.2, Vienna (Austria).

#### Network Visualization

The network analysis was performed via the creation of the network graphs using the ‘igraph’ package in R.^22^ A geographic layout with weighted edges was used to reflect the proportion of re-triages contributing to the specific edge from the total count of re-triages of seriously injured patients in the CA trauma system network in 2018. The force-directed algorithm, referred to as Fruchterman-Reingold, was used in all other visualizations. This algorithm relied on spring embedders that place center locations by assigning forces according to the edges (re-triage directions) connecting the centers.^23^

#### Optimization Estimations

The optimization algorithm consisted of 2 steps. In the first step, optimal re-triage was identified using the definition of seriously injured patient encounters presenting to nontrauma or level III/IV trauma centers then re-triaged to any level I or II trauma center within a day. Suboptimal re-triage was identified using the definition of a seriously injured patient presenting to nontrauma or level III/IV trauma centers and re-triaged to a second non-trauma or level III/IV center within a day.

In the second step, alternative optimal re-triage destinations were identified for all sub-optimal re-triages. Alternative optimal re-triage destinations were identified by finding the surrounding level I and II trauma centers and selecting the center with the shortest transport time as the optimal receiving center. Transport time was estimated from the drive and flight times between the sending and receiving trauma centers using center geolocation. The drive times between centers were calculated assuming the shortest driving path in the road network using the open-source routing machine API^24^ and average road speeds. The assumption was made that ground transport would be fastest at distances between centers <50 miles, and air transport would be fastest at distances ≥50 miles.^25–27^ Air transport is slower than ground transport over short distances because it depends on (i) the time to secure an air ambulance, (ii) additional ED-to-airport ground commute time, (iii) weather conditions that limit air transport speed, and (iv) air ambulance type (rotor versus fixed-wing). We could not account for these delays owing to the absence of transportation type details. Instead, we assumed the most conservative average air transport speed of 120 mph across a straight-line flight distance between the sending and receiving centers.

The alternative receiving destinations were considered from the list of operating level I or II trauma centers that year. When multiple alternative level I or II trauma centers were identified, the preference was given to the shortest ground transport time if the level I or II trauma center was identified within less than 50 miles. If there were no level I or II trauma centers within a driving distance of 50 miles, flight times to the closest level I or II were calculated and labeled as an optimal re-triage destination by air transport.

## RESULTS

The total number of encounters of seriously injured patients who were taken from the field to a nontrauma, level III, or level IV center during the study period was 43,066. A total of 34,184 (79.4%) encounters were not transferred to another hospital on the first day, thus, were under-triaged and not included in further analyses. A total of 8882 (20.6%) were transferred to another hospital within the first day and thus were labeled re-triaged. The number of re-triages increased from 698 in 2009 to 1209 in 2018 (see Table, Supplemental Table 2, http://links.lww.com/AOSO/A221). Most of the re-triages from sending centers (93.4 ± 2.6%) were admitted for inpatient care at receiving centers. On average, seriously injured patients were 66.27 ± 20.12 years old, predominantly male (59.2%), and white (74.3%) (Table 1).

**TABLE 1.** Characteristics of Re-triaged Seriously Injured Patients in California 2009–2018.

**Variable**	**Level**	**Re-triages N (%**)	**Sub-optimal Re-triages N (%**)	**Optimal Re-triages N (%**)
Patient Characteristics				
Total (N)		8882	2680	6202
Age, years	18–24	420 (4.7)	83 (3.1)	337 (5.4)
	25–34	521 (5.9)	88 (3.3)	433 (7.0)
	35–44	532 (6.0)	101 (3.8)	431 (6.9)
	45–54	811 (9.1)	143 (5.3)	668 (10.8)
	55–64	1253 (14.1)	327 (12.2)	926 (14.9)
	65–74	1479 (16.7)	482 (18.0)	997 (16.1)
	75–84	2133 (24.0)	794 (29.6)	1339 (21.6)
	≥85	1733 (19.5)	662 (24.7)	1071 (17.3)
Sex	Male	5258 (59.2)	1469 (54.8)	3789 (61.1)
	Female	3624 (40.8)	1211 (45.2)	2413 (38.9)
Race	White	6595 (74.3)	1800 (67.2)	4795 (77.3)
	Black	410 (4.6)	175 (6.5)	235 (3.8)
	Asian	620 (7.0)	313 (11.7)	307 (5.0)
	Other	1257 (14.2)	392 (14.6)	865 (13.9)
Ethnicity	Hispanic	1920 (21.6)	508 (19.0)	1412 (22.8)
	Non-Hispanic	6962 (78.4)	2172 (81.0)	4790 (77.2)
Insurance	Self-pay	627 (7.1)	91 (3.4)	536 (8.6)
	Blue Shield Blue Cross	284 (3.2)	28 (1.0)	256 (4.1)
	Private	371 (4.2)	95 (3.5)	276 (4.5)
	Medicare	5119 (57.6)	1784 (66.6)	3335 (53.8)
	Medicaid	990 (11.1)	152 (5.7)	838 (13.5)
	Federal	85 (1.0)	7 (0.3)	78 (1.3)
	Managed Care Unspecified	1163 (13.1)	490 (18.3)	673 (10.9)
	Other	243 (2.7)	33 (1.2)	210 (3.4)
Injury mechanism	All transport	1349 (15.2)	204 (7.6)	1145 (18.5)
	Fall	5713 (64.3)	1937 (72.3)	3776 (60.9)
	Struck by or against	506 (5.7)	134 (5.0)	372 (6.0)
	Other	1314 (14.8)	405 (15.1)	909 (14.7)
Body part affected	Torso	411 (4.6)	60 (2.2)	351 (5.7)
	Traumatic brain injury	7798 (87.8)	2470 (92.1)	5328 (85.9)
	Other head, face, and neck	255 (2.9)	31 (1.2)	224 (3.6)
	Upper/lower extremities	177 (2.0)	85 (3.2)	92 (1.5)
	Other	241 (2.7)	35 (1.3)	206 (3.3)
Injury Severity Score	16–25	8160 (91.9)	2543 (94.9)	5617 (90.6)
	>25	722 (8.1)	137 (5.1)	585 (9.4)
Sending center location				
Regional trauma Coordinating committee	South-East	2036 (22.9)	411 (15.3)	1625 (26.2)
	North	1882 (21.2)	414 (15.4)	1468 (23.7)
	Bay Area	1599 (18.0)	600 (22.4)	999 (16.1)
	Central	1814 (20.4)	188 (7.0)	1626 (26.2)
	South-West	1551 (17.5)	1067 (39.8)	484 (7.8)

**FIGURE 1.**
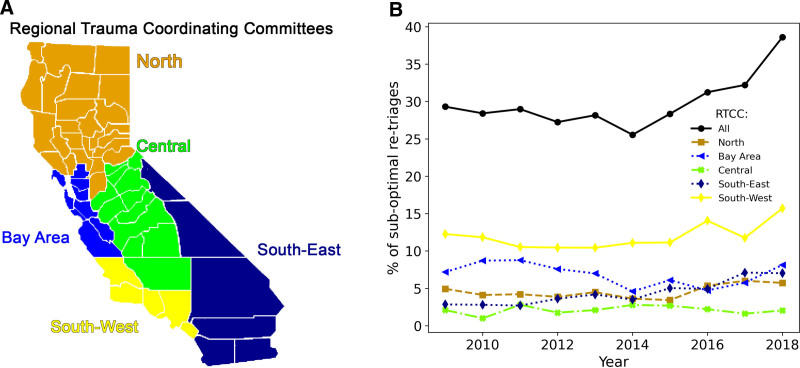
Regional Trauma Coordinating Committees Map (A) and suboptimal re-triage rate by year (B). LEMSA falls into unofficial, voluntary Regional Trauma Coordinating Committees (RTCC), as depicted in the map. Each RTCC accounts for a different proportion of all suboptimal re-triages, with Southwest accounting consistently for the largest proportion of suboptimal re-triages. The proportion of suboptimal re-triages accounted for by each RTCC, as denoted by the colored dashed lines, remained relatively constant over time. However, the overall statewide suboptimal re-triage rate, as denoted by the black solid line, increased over time.

During the entire study period, 2,680 (30.2%) re-triages in California were suboptimal or transferred to a second nontrauma center or level III/IV instead of a level I/II center. The suboptimal re-triage rate trended upward during the study period, from 29.3% in 2009 to 38.6% in 2018. Suboptimal re-triage was most frequently observed in RTCCs with the highest population density (Fig. 1). The sub-optimal re-triage rate in southwest RTCC was 68.8% compared to 10.2%–37.5% for all other RTCCs. The southwest RTCC accounted for 39.8% of all suboptimal re-triages in California.

The Los Angeles County LEMSA in the Southwest RTCC accounted for the largest proportion, 28.4% of sub-optimal re-triages in California during the entire study period (Supplemental Table 3 http://links.lww.com/AOSO/A221). Despite its high population, it accounted for only 10.2% of all re-triages in California (see Supplemental Table 3, http://links.lww.com/AOSO/A221). This is in stark contrast to Central California LEMSA, which accounted for 1.2% of all suboptimal re-triages but 11.3% of all re-triages in California during the entire study period. Each year during the study period, any single LEMSA accounted for 0%–13% of all suboptimal re-triages in any given RTCC (see Figure 1, http://links.lww.com/AOSO/A221).

**FIGURE 2.**
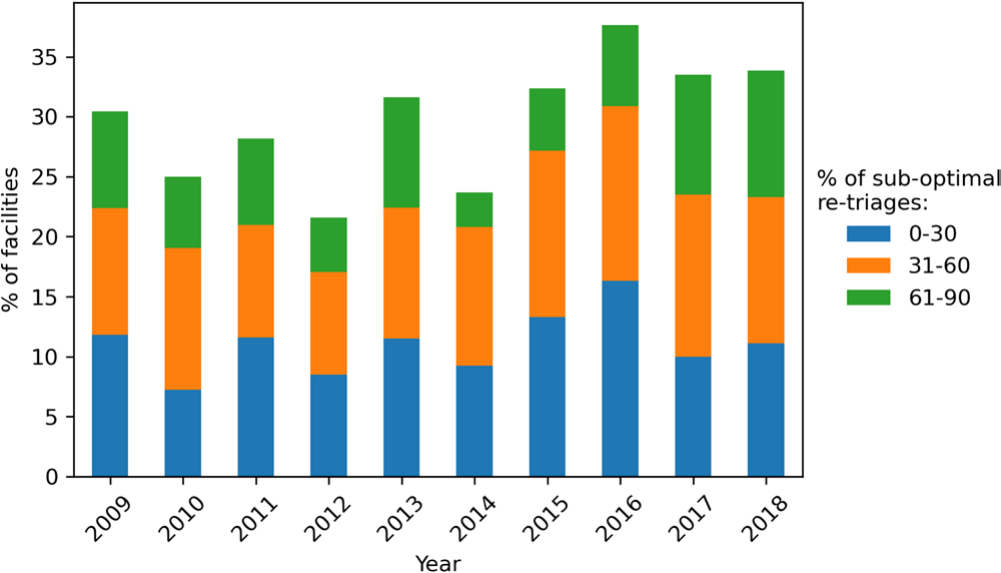
The Proportion of nontrauma or level III/IV trauma centers with at-least one suboptimal re-triage by year with center-level suboptimal re-triage rate. The proportion of nontrauma or level III/IV trauma centers with at least one suboptimal re-triage in each calendar year from 2009 to 2018. Stacked bar-graph colored segment denotes the center-level suboptimal re-triage rate (ranging from 10% to 90 % per figure legend).

During the entire study period, 56.0% of nontrauma or level III/IV trauma centers had at least one suboptimal re-triage. In any given year, 22%–37 % of nontrauma or level III/IV trauma centers had at least one suboptimal re-triage (Fig. 2). Every year, approximately 18% of sending nontrauma or level III/IV trauma centers suboptimally re-triaged ≥40% of all re-triaged seriously injured patients. Dense urban RTCC had less than 1 level I/II receiving center per 1,000,000 residents (see Supplemental Table 4, http://links.lww.com/AOSO/A221).

Generally, suboptimally re-triaged patients had lower rates of surgery (Table 2). However, the direction of the association between suboptimal re-triage flipped in high population density geographic areas. Specifically, South-West and Bay Area RTCC had higher rates of major and minor surgery among suboptimally re-triaged patients than optimally re-triaged patients in the same region. The length of stay in receiving centers was a median of 4 days for both suboptimal and optimal re-triages. Furthermore, suboptimally re-triaged severely injured patients had 1.53 times higher odds (95% confidence interval [CI] 1.23–1.90] of being discharged/transferred to a third short-term acute care general hospital for inpatient care than optimally re-triaged patients (See Supplemental Table 5, http://links.lww.com/AOSO/A221). Suboptimally seriously injured patients who survived to discharge had 1.25 higher odds of re-admission within 60 days than optimally re-triaged patients (see Supplemental Tables 9 and 10, http://links.lww.com/AOSO/A221).

**TABLE 2.** RTCC Surgery Rates Differ between Sub-optimal and Optimal Re-triaged Seriously Injured Patients in California 2009–2018

**Regional Trauma Coordinating Committee**	**Surgery Type** **^20,21^**	
	**Sub-optimal Re-triage** N (%)	**Optimal Re-triage** N (%)
Total N	2680	6202
	Minor therapeutic	
Bay Area	155 (5.8)	208 (3.4)
Central	33 (1.2)	316 (5.1)
North	81 (3.0)	300 (4.8)
South-East	97 (3.6)	373 (6.0)
South-West	187 (7.0)	96 (1.5)
	Major therapeutic	
Bay Area	249 (9.3)	288 (4.6)
Central	37 (1.4)	441 (7.1)
North	107 (4)	415 (6.7)
South-East	110 (4.1)	411 (6.6)
South-West	272 (10.1)	147 (2.4)
	Diagnostic	
Bay Area	31 (1.2)	37 (0.6)
Central	3 (0.1)	55 (0.9)
North	8 (0.3)	27 (0.4)
South-East	15 (0.6)	90 (1.5)
South-West	43 (1.6)	24 (0.4)

The GLMM model demonstrated (Table 3) that patients between 75 and 84 years old had 1.62 higher odds of sub-optimal re-triage (95% CI, 1.05–2.50) than seriously injured between 18 and 24 years old. Patients 85–89 years had 1.77 higher odds of suboptimal re-triage (95% CI, 1.13–2.76) than seriously injured patients between 18 and 24 years old. Females had 1.35 higher odds of suboptimal re-triage (95% CI, 1.17–1.57) compared to males. The odds of suboptimal re-triage were significantly higher for private (OR, 2.32; 9 5% CI, 1.48–3.62), Medicare (OR, 2.27; 95% CI, 1.54–3.36), and Managed Care unspecified (OR, 4.09; 95% CI, 2.78–6.00) insured patients, compared to self-pay patients. Patients with RISS ≥25 had 0.66 lower odds of sub-optimal re-triage (95% CI, 0.43–1.01) than patients with RISS less than or equal to 25. There were higher adjusted odds for sub-optimal re-triage for traumatic brain injuries (OR, 2.09; 95% CI, 1.39–3.15) and lower extremity injuries (OR, 7.72; 95% CI, 4.26–13.98) compared to torso injuries. Even after adjusting for clustering at the year and sending center, the South-West RTCC was significantly associated with 25.51 increased odds of sub-optimal re-triage (95% CI, 9.42–69.14).

**TABLE 3.** Predictors of Sub-optimal Re-triage in California from 2009 to 2018

**Variable**	**Level**	**Odds Ratio**	***P*-value**
Age, years (vs. 18–24)			
	25–34	1.17 (0.72–1.90)	0.5
	35–44	0.99 (0.61–1.59)	1
	45–54	0.75 (0.48–1.18)	0.2
	55–64	1.11 (0.73–1.68)	0.7
	65–74	1.38 (0.89–2.13)	0.1
	75–84	1.63 (1.06–2.53)	0.03*
	≥85	1.77 (1.13–2.76)	0.01*
Sex (vs. male)			
	Female	1.35 (1.17–1.57)	<0.001***
Race (vs. White)			
	Black	0.85 (0.60–1.23)	0.4
	Asian	0.92 (0.69–1.23)	0.6
	Other	0.80 (0.62–1.04)	0.1
Ethnicity (vs. non-Hispanic)			
	Hispanic	0.95 (0.76–1.20)	0.7
Insurance (vs. self-pay)			
	Private	2.32 (1.48–3.62)	<0.001***
	Medicare	2.27 (1.54–3.36)	<0.001***
	Medicaid	1.05 (0.70–1.60)	0.8
	Managed Care Unspecified	4.09 (2.78–6.00)	<0.001***
	Other	1.09 (0.62–1.93)	0.8
Injury mechanism (vs. all transport)			
	Fall	1.98 (1.51–2.60)	<0.001***
	Struck by or against	2.41 (1.63–3.58)	<0.001***
	Other	1.98 (1.46–2.68)	<0.001***
Body part affected (vs. torso)			
	Traumatic brain injury	2.09 (1.39–3.15)	<0.001***
	Other head, face, and neck	0.95 (0.50–1.82)	0.9
	Upper/lower extremities	7.72 (4.26–13.98)	<0.001***
	Other	0.93 (0.48–1.78)	0.8
Injury Severity Score (vs. 16–25)			
	>25	0.66 (0.43–1.01)	0.06
Regional Trauma Coordinating Committee (vs. Central)			
	North	1.35 (0.46–3.92)	0.6
	Bay Area	7.11 (2.43–20.81)	<0.001***
	South-East	2.54 (0.85–7.61)	0.09
	South-West	25.51 (9.42–69.14)	<0.001***

*P values and Confidence Intervals generated from a generalized linear mixed-effects (GLMM) regression modeling the probability of suboptimal re-triage was used to quantify the association with RTCC of the sending center while controlling for a priori determined fixed-effect predictor variables including age, sex, race, ethnicity, insurance status, RISS, injury mechanism, and random effects to control for clustering by sending center and year.

* indicates *p*<0.05.

*** indicates *p*<0.001.

**FIGURE 3.**
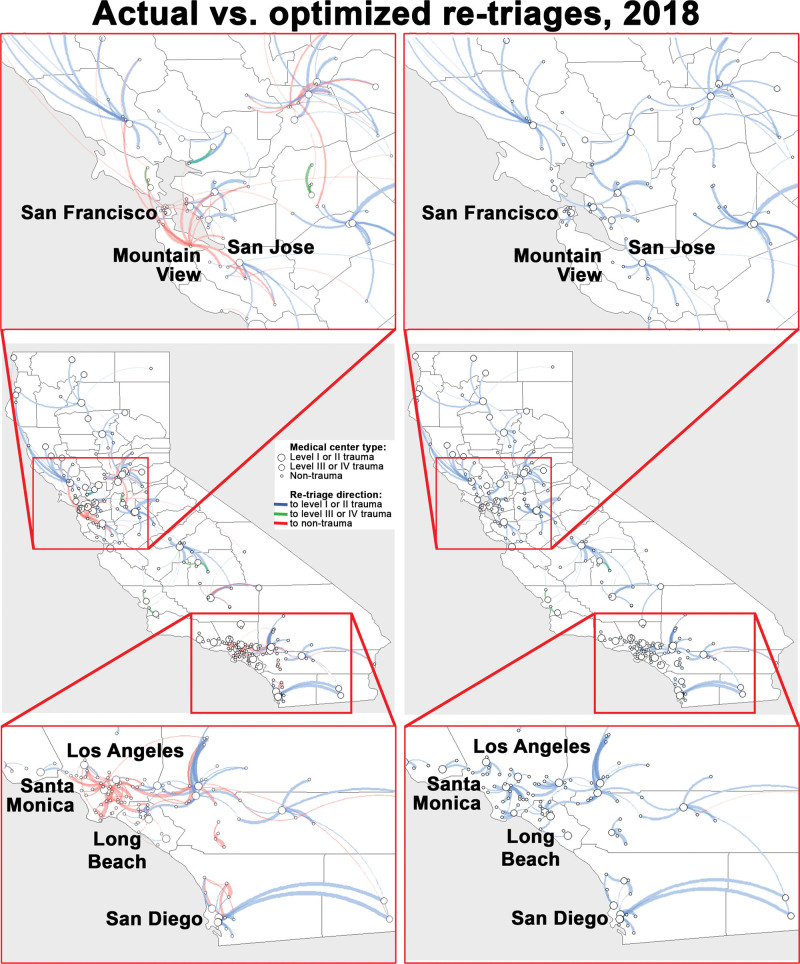
Actual (left panel) vs. Optimized (right panel) re-triages in 2018 in California’s trauma system. The re-triage volumes between centers were reflected by the thickness of the lines connecting the medical centers. The blue lines show optimal re-triages to level I or II trauma, the red lines show suboptimal re-triages between nontrauma centers, and the green lines show suboptimal re-triages between nontrauma centers and level III or IV trauma centers.

California Trauma Network Performance in 2018 was mapped. The entire statewide network-level rate of suboptimal re-triage was 38.6%, with the LEMSAs neighboring San Francisco and Los Angeles contributing the largest proportion (Fig. 3, left panel). Our algorithm identified an alternative optimal re-triage receiving center for 36.8% out of 38.6% of sub-optimal re-triages (Fig. 3, right panel).

## DISCUSSION

Timely re-triage of seriously injured patients can reduce the mortality associated with under-triage.^28,29^ Patient characteristics associated with failure to re-triage have been well described.^30^ Previously, we have identified receiving trauma center acceptance as a major barrier to re-triage in prior failure modes effects analysis work.^31^ When we considered where re-triaged patients were transported, we saw RTCC as most highly associated with suboptimal re-triage. This study demonstrated that suboptimal re-triages were concentrated in RTCCs with dense urban areas, such as the southwest. We found that suboptimally re-triaged patients had higher rates of transfer to a third short-term general hospital and increased rates of re-admission within 10, 30, and 60 days from discharge. All RTCCs had increased suboptimal re-triage rates over time, except for the most rural Central RTCC. The network analysis demonstrated that suboptimal functioning segments could be visualized, and geospatial optimization algorithms could successfully find alternative optimal re-triage destinations for most suboptimal re-triages.

Similar to Gomez et al.^32^ our findings demonstrate that dense urban areas with less of receiving centers and more sending centers account for a larger proportion of suboptimal re-triage statewide and have a higher suboptimal re-triage rate. The low rate of sub-optimal re-triage in remote nontrauma and level III/IV centers was most likely due to the lack of alternative receiving centers.^33,34^ Well-defined LEMSA re-triage guidelines, such as in the Central RTCC, may have also contributed to reducing sub-optimal re-triage.^35,36^

The American College of Surgeons Committee on Trauma has recognized the imbalance in trauma system functioning and attempted to address it by introducing the Needs-Based Assessment of Trauma Systems (NBATS) tool.^37,38^ The tool relies on expert consensus guidelines for specific geographic areas to optimize the number and location of trauma centers. Although the usage of such a tool is reasonable for underdeveloped trauma systems, it may be less useful in the mature trauma system. NBATS cannot differentiate how the trauma system (including high-level, low-level, and non-trauma centers) functions together to accomplish the task of caring for all injured patients. Instead, NBATS considers only the volume of severely injured patients treated at nontrauma centers and the difference between the observed and expected volume of severely injured patients at level I/II trauma centers in each trauma service area. NBATS assumes that the only reason severely injured patients would be treated at nontrauma centers or that there would be a sizeable discrepancy between observed and expected volume of severely injured at high-level trauma centers, is a lack of high-level trauma center bed availability. Ours and prior work have demonstrated that this is an incorrect assumption.^31,39,40^ NBATS commonly signals a need for extra trauma centers in rural areas and fewer in urban areas regardless of how the trauma system functions. Most concerning, these recommendations are rarely practically actionable. Our current study identified that rural areas function well and large, urban areas have the greatest opportunity for improvement of re-triage optimality. We believe geospatial analyses in trauma care should be expanded toward creating human-machine interaction tools to optimize the performance of the entire trauma system by optimally leveraging all existing resources to manage the load of injured patients. Further tool development could be integrated with EMS and at the state level to improve trauma system functioning in real time. We believe that a “buddy” or partnering concept, whereby nontrauma and level III/IV centers send to a specific single high-level receiving center, may also optimize re-triage in poorly coordinated urban areas. Improving coordination with real-time geospatial calculations may ensure ideal resource utilization, reduce re-triage time,^41^ and ultimately reduce injury-associated mortality.^42^ Such tools have been demonstrated in previous stroke literature to help find suitable alternatives when transferring to a higher level of care.^43^

## LIMITATIONS

The current study had several limitations. First, we identified re-triage by linking administrative data using a unique RLN (see the description above). Before the linkage, we identified that approximately 25% of ED encounters and 11%–14% of inpatient hospitalization encounters were missing unique identifiers required to link encounters during re-triage. This may have introduced a selection bias where re-triages of some seriously injured patients (e.g., undocumented people without social security numbers used to derive the dummy unique patient identifier) were not included in our analyses (see Supplemental Table 11, http://links.lww.com/AOSO/A221 for details). Second, the administrative data sources had limited clinical data.^44,45^ This may have introduced unmeasured variable bias because we were not able to include clinical variables commonly used to risk adjust in trauma, such as admission blood pressure, pulse, or Glasgow Coma Score. We addressed this by estimating the RISS^10^ and including the injury mechanism in the multivariable model. In addition, the definition of suboptimal re-triage was limited by the fact that it was not possible to estimate re-triage time because discharge time, admission time, and transport type data were not available. Defining the re-triage time with assumptions of traffic patterns, weather, and the availability of transport mechanisms would have been imprecise. Therefore, the most conservative definition of suboptimal re-triage was applied to minimize bias. Finally, a possible motivation for sub-optimal re-triage might be dictated by the availability of the required subspecialty in the closest level III/IV trauma center. Testing this hypothesis was outside of the scope of these data.

## CONCLUSION

Our analyses demonstrated that RTCCs with large urban LEMSAs accounted for the largest proportion of suboptimal re-triages in California. They also had the highest suboptimal re-triage rate. Conversely, rural Central RTCC accounted for the largest proportion of re-triage and had the lowest suboptimal re-triage rate. The observed localized suboptimality in California’s trauma system shows an opportunity for improvement where network visualization and optimization estimation methods could be used to optimize re-triage.

## Supplementary Material


