# Supplementary material for: Geographic Disparities in Re-triage Destinations Among Seriously Injured Californians
Source: Ann Surg Open. 2023 Mar 17;4(1):e270. doi: 10.1097/AS9.0000000000000270 (PMC10348777; doi:10.1097/AS9.0000000000000270)
Supplement: Supplementary file 1 [file as9-4-e270-s001.pdf]

Supplemental Digital Content for
Geographic Disparities in Re-triage Destinations Among Seriously Injured Californians

Al'ona Furmanchuk,[a,b*] PhD, (alona.furmanchuk@northwestern.edu)
Kelsey James Rydland,[c] PhD, (Kelsey.rydland@northwestern.edu)
Renee Y. Hsia[d,e], MD, (renee.hsia@ucsf.edu)
Robert Mackersie,[f] MD, FACS (robert.mackersie@ucsf.edu)
Meilynn Shi, [a] BS (Meilynn.shi@northwestern.edu)
Mark William Hauser,[g] PhD, (mark.hauser@northwestern.edu)
Abel Kho, [a,b] MD, MS, FACMI (akho@nm.org)
Karl Y. Bilimoria,[h] MD, MS, FACS (karl.bilimoria@northwestern.edu)
Anne M. Stey,[h] MD, MSc, FACS (anne.stey@northwestern.edu)

[a] Northwestern University Feinberg School of Medicine, Division of General Internal Medicine and Geriatrics, Chicago, IL;
[b] Center for Health Information Partnerships (CHiP), Institute for Public Health and Medicine, Northwestern University Feinberg School of Medicine, Chicago, IL, USA, Chicago, IL;
[c] Northwestern University Libraries, Research and Data Services, Evanston, IL;
[d] University of California San Francisco, Department of Emergency Medicine, San Francisco, CA;
[e] Philip R. Lee Institute for Health Policy Studies, University of California San Francisco, San Francisco, CA
[f] University of California San Francisco, Department of Surgery, San Francisco, CA;
[g] Northwestern University, Department of Anthropology, Evanston, IL;
[h] Northwestern University Feinberg School of Medicine, Department of Surgery, Chicago, IL

Content

Supplemental Digital Content 1. Table 1. illustrates data management is used to produce a universe of relevant re-triages and sub-optimal re-triages.
Supplemental Digital Content 2. Table 2. illustrates annual counts of re-triages and under-triages in the California Department of Healthcare Access and Information (HCAI) ED and inpatient discharge datasets. Re-triages are identified according to the direction: from ED department of sending facility to the inpatient settings of receiving facility (ED-to-PDD) and between ED departments of different facilities (ED-to-ED).
Supplemental Digital Content 3. **Table 3.** illustrates re-triages by Sending Center LEMSA of Seriously Injured Patients in California from 2009-2018.
Supplemental Digital Content 4. Figure 1. illustrates annual sub-optimal re-triages of Seriously Injured Patient in California from 2009-2019 in each local emergency medical services agencies (LEMSA)segregated by regional trauma coordination center (RTCC)
Supplemental Digital Content 5. Table 4. illustrates **c**apacity and demand of trauma care by RTCC.
Supplemental Digital Content 6. Table 5. illustrates the length of stay and discharge disposition in receiving center from sub-optimal versus optimal re-triaged seriously injured patients in California 2009-2018.
Supplemental Digital Content 6. Table 6. illustrates unadjusted rates of discharge disposition from sub-optimal versus optimal re-triaged seriously injured patients in California 2009-2018.
Supplemental Digital Content 6. Table 7. illustrates the odds ratio estimated (using logistic regression) for expected discharge disposition outcome after sub-optimally re-triaged versus optimally re-triaged patients
Supplemental Digital Content 6. Table 8. illustrates **v**alue sets used to group discharge disposition (disp) variable into categories.
Supplementary Digital Content 7. Table 9. Illustrates re-admission after discharge from sub-optimal versus optimal re-triage destination among seriously injured patients in California 2009-2018.

Supplementary Digital Content 7. Table 10. illustrates the odds ratio estimated (using logistic regression) for re-admission after discharge from sub-optimal versus optimal re-triage destination among seriously injured patients in California 2009-2018.

Supplementary Digital Content 8. Table 11. illustrates an annual summary on severely injured patients listed as transferred but not subsequently found in a second hospital.

Supplemental Digital Content 1. Table 1. Data management is used to produce a universe of relevant re-triages and sub-optimal re-triages.

**Data management**	**Step**	**Processing logic**	**Total records**
Pre-processing	1	Records from period 2009-2018 were collected	ED: 41224883<br><br>IP: 7,439,260
	2	Unique records produced using all fields available	ED: 24,592,353<br><br>IP: 4,036,917
Relevant ED record collection or starting point of the re-triage	3	ED records for patient of age 18-89 years old having valid record linkage number available<br>**AND** RISS>15<br>**AND** presented to a non-trauma or level III/IV trauma centers with injury diagnosis defined by the ICD-9: 800-904.9, 910-939.0, and 950-959.9 or ICD-10: S00-T19, T33-T34, T79 codes<br>**AND** discharge dispositions not ‘Expired’, ‘Left against medical advice or discontinued care’, or ‘Discharged/Transferred to an Inpatient Rehabilitation Facility (IRF) including Rehabilitation Distinct Part Unit of a Hospital with a Planned Acute Care Hospital Inpatient Readmission’.	55,222
Denominator or universe of all relevant re-triages	4	Records after ED discharges where linked (using record linkage number) to inpatient admission record except for Emergency or Elective type inpatient admissions. Admissions up to 1 day after discharge were considered.	8,882
Numerator or sub-optimal re-triages	5	Records with inpatient destination being level III/IV trauma and non-trauma centers.	2,680

Supplemental Digital Content 2. Table 2. Annual counts of re-triages and under-triages in the California Department of Healthcare Access and Information (HCAI) ED and inpatient discharge datasets. Re-triages are identified according to the direction: from ED department of sending facility to the inpatient settings of receiving facility (ED-to-PDD) and between ED departments of different facilities (ED-to-ED).

**Triage direction**	**Year**													
	**2005**	**2006**	**2007**	**2008**	**2009**	**2010**	**2011**	**2012**	**2013**	**2014**	**2015**	**2016**	**2017**	**2018**
Total re-triages*:	226	487	518	653	698	640	896	838	1002	899	1003	1062	1307	1209
ED-to-PDD	218	464	486	618	669	609	856	804	959	830	935	954	1183	1083
ED-to-ED	8	23	32	35	29	31	40	34	43	69	68	108	124	126
Total under-triages**	2474	2540	2924	2795	3025	2990	2853	3027	3243	3264	3509	5257	3373	3643
Total triages	2692	3004	3410	3413	3694	3599	3709	3831	4202	4094	4444	6211	4556	4726

*Re-triage was defined as a patient with a RISS>15 who first visited a non-trauma or Level I/II trauma center, but was subsequently taken to another center.

**Under-triage was defined as any patient with an ISS>15 who was never taken to a Level I/II trauma center.

The under-triaged patients are patients that were never re-triaged from the original non-trauma or level III/IV trauma center.

Supplemental Digital Content 3. **Table 3.** Re-triages by Sending Center LEMSA of Seriously Injured Patients in California from 2009-2018.

Sending center location by LEMSA	Re-triages<br>N=8,882 (%)	Sub-optimal re-triages<br>N=2,680 (%)	Optimal re-triages<br>N=6,202 (%)
**North Region**			
Coastal Valleys	385 (4.3)	51 (1.9)	334 (5.4)
El Dorado County	40 (0.5)	15 (0.6)	25 (0.4)
Napa County	63 (0.7)	8 (0.3)	55 (0.9)
NorCal	30 (0.3)	0 (0)	30 (0.5)
North Coast	292 (3.3)	44 (1.6)	248 (4)
Sacramento County	113 (1.3)	24 (0.9)	89 (1.4)
San Joaquin County	352 (4)	139 (5.2)	213 (3.4)
Sierra-Sacramento	570 (6.4)	113 (4.2)	457 (7.4)
Yolo County	37 (0.4)	20 (0.7)	17 (0.3)
**Bay Area Region**			
Alameda County	471 (5.3)	201 (7.5)	270 (4.4)
Contra Costa County	475 (5.3)	89 (3.3)	386 (6.2)
Marin County	59 (0.7)	55 (2.1)	4 (0.1)
Monterey County	47 (0.5)	15 (0.6)	32 (0.5)
San Benito County	36 (0.4)	6 (0.2)	30 (0.5)
Santa Clara County	127 (1.4)	63 (2.4)	64 (1)
Santa Cruz County	33 (0.4)	9 (0.3)	24 (0.4)
San Francisco County	108 (1.2)	65 (2.4)	43 (0.7)
San Mateo County	51 (0.6)	44 (1.6)	7 (0.1)
Solano County	192 (2.2)	53 (2)	139 (2.2)
**Central Region**			
Central California	1006 (11.3)	31 (1.2)	975 (15.7)
Kern County	199 (2.2)	113 (4.2)	86 (1.4)
Merced County	175 (2)	2 (0.1)	173 (2.8)
Mountain Valley	273 (3.1)	36 (1.3)	237 (3.8)
Tuolumne County	161 (1.8)	6 (0.2)	155 (2.5)
**South-East Region**			
Inland Counties	525 (5.9)	108 (4)	417 (6.7)
Imperial County	293 (3.3)	8 (0.3)	285 (4.6)

Riverside County	549 (6.2)	220 (8.2)	329 (5.3)
San Diego County	669 (7.5)	75 (2.8)	594 (9.6)
**South-West Region**			
Los Angeles County	903 (10.2)	760 (28.4)	143 (2.3)
Orange County	326 (3.7)	107 (4)	219 (3.5)
San Luis Obispo County	165 (1.9)	145 (5.4)	20 (0.3)
Santa Barbara County	68 (0.8)	2 (0.1)	66 (1.1)
Ventura County	89 (1)	53 (2)	36 (0.6)

Supplemental Digital Content 4. Figure 1. Annual sub-optimal re-triages of Seriously Injured Patient in California from 2009-2019 in each local emergency medical services agencies (LEMSA)segregated by regional trauma coordination center (RTCC)

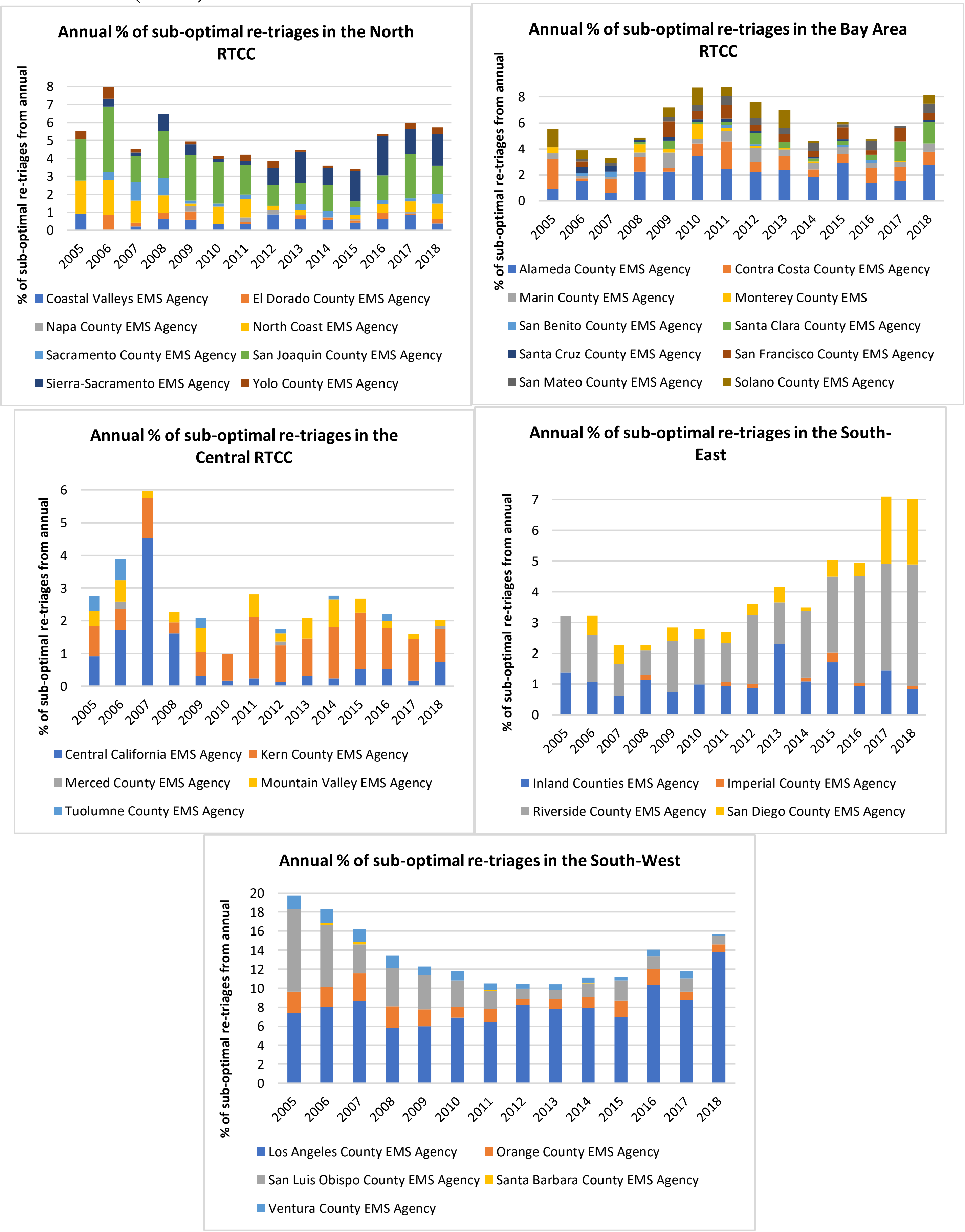

Supplemental Digital Content 5. Table 4. Capacity and demand of trauma care by RTCC.

RTCC	The total population during 2009-2018, Average ± SD	The annual average number of Level I/II trauma centers	The annual average number of Level III/IV & non-trauma	Level I/II trauma centers per 1,000,000 residents	Level I/II trauma centers per 100 Level III/IV & non-trauma
North	4,787,666 ± 110,911	6.0	85.5	1.25	7.0
Bay Area	7,558,614 ± 247,741	6.4	85.3	0.85	7.5
Central	3,541,876 ± 93,750	4.0	50.6	1.13	7.9
South-East	7,793,012 ± 241,303	8.8	71.8	1.13	12.3
South-West	14,708,537 ± 290,637	12.1	162.6	0.82	7.4

Supplemental Digital Content 6. Table 5. The length of stay and discharge disposition in receiving center from sub-optimal versus optimal re-triaged seriously injured patients in California 2009-2018.

Re-triage	Total N	Length of stay, days	
		Average ± Standard Deviation	Median (Interquartile Range)
Sub-optimal	2677	11.2 ± 15.3	4 (1-13.5)
Optimal	6205	16.2 ± 26.9	4 (1-15.5)

Supplemental Digital Content 6. Table 6. Unadjusted rates of discharge disposition from sub-optimal versus optimal re-triaged seriously injured patients in California 2009-2018.

Re-triage	Discharge disposition n(%)				
	Total N	Home	Died	Short-term Acute Care Hospital	Post-acute care
**Sub-optimal**	2680 (100)	1650 (61.6)	164 (6.1)	141 (5.3)	725 (27.1)
**Optimal**	6202 (100)	3794 (61.2)	410 (6.6)	217 (3.5)	1781 (28.7)

Supplemental Digital Content 6. Table 7. Odds ratio estimated (using logistic regression) for expected discharge disposition outcome after sub-optimally re-triaged versus optimally re-triaged patients

Discharge Disposition of Re-triaged	Odds Ratio	Std Err	p-value	[95% Conf. Interval]
Home*	1.02	0.05	0.727	[0.93 - 1.12]
Died*	0.92	0.09	0.387	[0.76 - 1.11]
Short-term Acute Care Hospital*	**1.53**	0.17	**<0.0001**	[1.23 - 1.90]
Post-acute care*	0.92	0.05	0.110	[0.83- 1.02]

*See **Supplemental Digital Content 6 Table 8** for the definition of the category.

Supplemental Digital Content 6. Table 8. Value sets used to group discharge disposition (disp) variable into categories.

Value sets for the period 2009-2014	Value sets for the period 2015-2018
**Home**	
12 = Home Health Service	01 = Discharged to home or self-care (routine discharge)
01 = Routine (home)	06 = Discharged/transferred to home under the care of an organized home health service organization in anticipation of covered skilled care
08 = Residential Care Facility	50 = Hospice - Home
	51 = Hospice - Medical facility (certified) providing hospice level of care
	81 = Discharged to home or self-care with a planned acute care hospital inpatient readmission
	65 = Discharged/transferred to a psychiatric hospital or psychiatric distinct part unit of a hospital

10 = Left Against Medical Advice<br>09 = Prison/Jail	07 = Left against medical advice or discontinued care<br>21 = Discharged/transferred to court/law enforcement<br>43 = Discharged/transferred to a federal healthcare facility
**Died**	
11 = Died	20 = Expired
**Short-term Acue Hospital**	
02 = Acute Care within the admitting hospital<br>05 = Acute Care at another hospital	02 = Discharged/transferred to a short-term general hospital for inpatient care<br>82 = Discharged/transferred to a short-term general hospital for inpatient care with a planned acute care hospital inpatient readmission
**Post-acute care**	
06 = Other Care (not SN/IC) at another hospital<br>03 = Other Care within the admitting hospital	90 = Discharged/transferred to an inpatient rehabilitation facility (IRF) including rehabilitation distinct part unit of a hospital with a planned acute care hospital inpatient readmission<br>70 = Discharged/transferred to another type of health care institution not defined elsewhere in this code list<br>04 = Discharged/transferred to a facility that provides custodial or supportive care (includes Intermediate Care Facility)<br>05 = Discharged/transferred to a designated cancer center or children's hospital<br>62 = Discharged/transferred to an inpatient rehabilitation facility (IRF) including a rehabilitation distinct part unit of a hospital<br>63 = Discharged/transferred to a Medicare-certified long-term care hospital (LTCH)<br>66 = Discharged/transferred to a Critical Access Hospital (CAH)<br>61 = Discharged/transferred to a hospital-based Medicare-approved swing bed
07 = Skilled Nursing / Intermediate Care (SN/IC) at another facility<br>04 = Skilled Nursing / Intermediate Care (SN/IC) within the admitting hospital	83 = Discharged/transferred to a skilled nursing facility (SNF) with Medicare certification with a planned acute care hospital inpatient readmission<br>03 = Discharged/transferred to a skilled nursing facility (SNF) with Medicare certification in anticipation of skilled care<br>64 = Discharged/transferred to a nursing facility certified under Medicaid (Medi-Cal), but not certified under Medicare

Supplementary Digital Content 7. Table 9. Re-admission after discharge from sub-optimal versus optimal re-triage destination among seriously injured patients in California 2009-2018.

**Survivor Re-admission**	**total N**	**Re-admission, n (%)**		
		**<=10 days**	**<=30 days**	**<=60 days**
Sub-optimal	2584	168 (6.5)	273 (10.6)	326 (12.6)
Optimal	5933	305 (5.1)	516 (8.7)	615 (10.4)

Supplementary Digital Content 7. Table 10. Odds ratio estimated (using logistic regression) for re-admission after discharge from sub-optimal versus optimal re-triage destination among seriously injured patients in California 2009-2018.

**Re-admission**	**Odds Ratio**	**Std Err**	**p-value**	**[95% Conf. Interval]**
≤10 days	1.28	0.13	0.012	[1.06 - 1.56]
≤30 days	1.24	0.10	0.006	[1.06 – 1.45]
≤60 days	1.25	0.09	0.002	[1.08 – 1.44]

Supplementary Digital Content 8. Table 11. Annual summary on severely injured patients listed as transferred but not subsequently found in a second hospital.

Year	Patients listed as transferred but not subsequently found in a second hospital
2009	202
2010	205
2011	233
2012	236
2013	236
2014	249
2015	216
2016	153
2017	390
2018	387
